# Deformation of a Red Blood Cell in a Narrow Rectangular Microchannel

**DOI:** 10.3390/mi10030199

**Published:** 2019-03-21

**Authors:** Naoki Takeishi, Hiroaki Ito, Makoto Kaneko, Shigeo Wada

**Affiliations:** 1 Graduate School of Engineering Science, Osaka University, 1-3 Machikaneyama, Toyonaka, Osaka 560-8531, Japan; shigeo@me.es.osaka-u.ac.jp; 2 Department of Mechanical Engineering, Osaka University, Suita, Osaka 565-0871, Japan; ito@hh.mech.eng.osaka-u.ac.jp (H.I.); mk@mech.eng.osaka-u.ac.jp (M.K.); 3 Department of Physics, Graduate School of Science, Chiba University, Chiba 263-8522, Japan

**Keywords:** red blood cells, Lattice–Boltzmann method, finite element method, immersed boundary method, narrow rectangular microchannel, computational biomechanics

## Abstract

The deformability of a red blood cell (RBC) is one of the most important biological parameters affecting blood flow, both in large arteries and in the microcirculation, and hence it can be used to quantify the cell state. Despite numerous studies on the mechanical properties of RBCs, including cell rigidity, much is still unknown about the relationship between deformability and the configuration of flowing cells, especially in a confined rectangular channel. Recent computer simulation techniques have successfully been used to investigate the detailed behavior of RBCs in a channel, but the dynamics of a translating RBC in a narrow rectangular microchannel have not yet been fully understood. In this study, we numerically investigated the behavior of RBCs flowing at different velocities in a narrow rectangular microchannel that mimicked a microfluidic device. The problem is characterized by the capillary number Ca, which is the ratio between the fluid viscous force and the membrane elastic force. We found that confined RBCs in a narrow rectangular microchannel maintained a nearly unchanged biconcave shape at low Ca, then assumed an asymmetrical slipper shape at moderate Ca, and finally attained a symmetrical parachute shape at high Ca. Once a RBC deformed into one of these shapes, it was maintained as the final stable configurations. Since the slipper shape was only found at moderate Ca, measuring configurations of flowing cells will be helpful to quantify the cell state.

## 1. Introduction

It is well known that many blood-related diseases are associated with alterations in the geometry and membrane properties of red blood cells (RBCs) that result in reduced functionality [1]. For instance, RBCs in patients with diabetes mellitus exhibit impaired cell deformability [2], as do those in patients with sepsis [3]. As another example, malaria-infected RBCs demonstrate membrane stiffening as well as shape distortion [4,5,6]. Hence, cell deformability may be an important indicator of cell state, and might be used to diagnoses relevant blood diseases. To date, various experimental techniques have been proposed to evaluate RBC deformability, e.g., optical tweezers and atomic force microscopy, but they usually suffer from low throughput. Recently, several microfluidic techniques that are capable of high-throughput measurement have been developed [7,8,9,10,11]. For instance, Ito et al. (2017) successfully developed a novel high-throughput assay to quantify the mechanical response of RBCs after spatial constriction, and found a characteristic mechanical response to long-term deformation that may have been related to chemical energy content [9].

Along with these experimental studies, recent computer simulation techniques have successfully been used to investigate aspects of cell dynamics such as stresses, velocities, and deformations, and have been shown to reproduce single-cell dynamics [12,13,14]. Mokbel et al. (2017) quantitatively related cell deformation to mechanical parameters in an experiment involving microfluidic flow through a square channel [13]. To elucidate patient-specific blood rheology, RBCs in diabetes mellitus and sickle-cell anemia were modeled in terms of cell rigidity and membrane viscosity, and their hydrodynamic interactions were quantified [15,16]. Since such numerical models allow us to investigate cell behavior in large parameter spaces, the coupling of experimental and numerical approaches may constitute a usefull bioengineering strategy to quantify the cell state.

Despite the studies referred to above, much is still unknown about the behavior of flowing RBCs, especially in a confined microchannel or between two closely spaced parallel plates (i.e., Hele-Shaw cell). Since the deformation of a RBC in a narrow rectangular microchannel is limited to an almost two-dimensional space, it is relatively easy to quantify the deformed configuration [17,18]. Although a number of studies using microfluidic devices have reported cellular-scale dynamics [19,20,21,22,23,24] as well as numerical studies [20,25,26,27,28], the dynamics of a translating RBC in a narrow rectangular microchannel have not yet been fully investigated. Recent our developed on-chip feedback manipulation system allowed us to investigate the two-dimensionally projected shape profile of RBCs, and showed RBC heterogeneity in a narrow rectangular microchannel [17,18]. However, a precise deformation especially in thickness direction of RBCs cannot be captured by means of the experimental observation.

One of the pioneering theoretical works about the behavior of the cell membrane in a confined channel was reported by Secomb & Skalak (1982) [29]. More recently, Tahiri et al. (2013) systematically investigated the shape transition of confined RBCs modeled as vesicles, and showed a phase diagram of the mode of RBCs [28]. Since these works were limited to the two-dimensional behavior of RBCs, it is unknown whether their insights are applicable to estimating the three-dimensional deformation of a RBC in a narrow rectangular microchannel. Fedosov et al. (2014) systematically investigated the behavior of a single RBC in cylindrical microchannels for a wide range of channel confinements (2a/D, being the radius of the RBC *a* and the channel diameter *D*) using a three-dimensional dissipative particle dynamics model [30]. However, their microchannels (2a/D< 0.8) had relatively large cross-sectional area comparing to a narrow rectangular microchannel represented in [17,18,31]. Zhu et al. (2016) numerically investigated the behavior of a droplet in a Hele-Shaw cell, and identified characteristic flow structures that were induced by the translating droplet [31]. Since forces acting on an interface depend on the constitutive law, it is expected that the hydrodynamic interaction between the fluid and cell membrane will differ from that observed in the droplet model.

The objective in this study, therefore, is to clarify the detailed behavior of translating RBCs in a narrow rectangular microchannel. The RBCs was modeled as a biconcave capsules, whose membranes followed the Skalak constitutive law [32]. We quantified the stable configuration of deformed RBCs in a narrow rectangular microchannel, mimicking a microfluidic device [17], for different values of the capillary number Ca, which is the ratio between the fluid viscous force and the membrane elastic force. We also investigated the effect on this configuration of altering parameters such as bending rigidity and viscosity ratio. To accelerate numerical simulations, we resorted to computing with a graphics processing unit (GPU), using the Lattice–Boltzmann method (LBM) for the inner and outer fluids and the finite element method to follow the deformation of the RBC membrane. These models were previously successfully applied to the analysis of cellular hydrodynamic interactions in channel flows [12,33,34,35].

## 2. Materials and Methods

### 2.1. Flow and RBC Model

**Figure 1:**
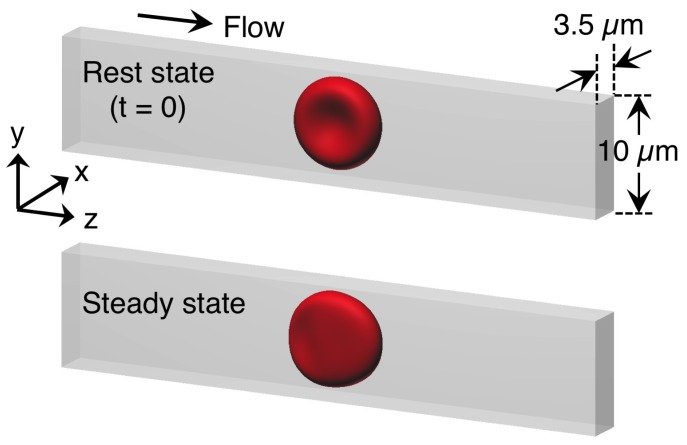
Computational domain to reproduce a translating red blood cell (RBC) in the narrow rectangular microchannel. The domain mimicked a microfluidic device as shown in Figure A1. The domain cross-section was 10 μm × 3.5 μm along the wall-normal and span-wise directions, respectively, and the stream-wise distance was set to be 50 μm. Flow direction is from left to right.

**Figure A1:**
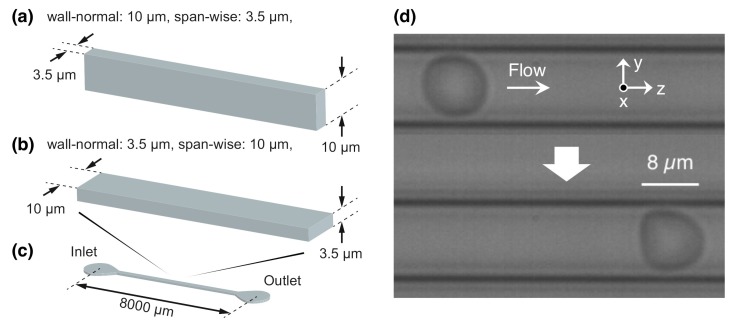
Detailed channel geometry. Magnified view of the channels with the cross-sections of (**a**) 10 μm × 3.5 μm and (**b**) 3.5 μm × 10 μm, which were used in Figure A2 and Figure A1, respectively. (**c**) Schematic view of the whole channel, whose stream-wise length was 8000 μm. (**d**) Representative images of a flowing RBC in a microfluidic device. Flow direction is from left to right.

We considered a cellular flow consisting of an external/internal fluid and a RBC membrane with radius *a* in a rectangular box representing a microfluidic device with 10 μm × 3.5 μm along the wall-normal and span-wise directions (Figure A1a). Representative images of a flowing RBC in a microfluidic device (Figure A1b,c) are shown in Figure A1d. The stream-wise distance for the computational domain was set to be 50 μm (Figure 1). Each RBC was modeled as a biconcave capsule, or a Newtonian fluid enclosed by a thin elastic membrane, with a major diameter 8 μm (=2*a*) and maximum thickness 2 μm (= *a*/2 = $t^{R}$). The flow was driven by a pressure gradient. Periodic boundary conditions were imposed on the inlet and outlet. To reproduce in vivo human RBC condition experimentally, the cytoplasmic viscosity was taken to be $\mu_{1}$ = 6.0 × 10${}^{-3}$ Pa·s, which is five times higher than the external fluid viscosity, $\mu_{0}$ = 1.2 × 10${}^{-3}$ Pa·s. Hence, the viscosity ratio λ (=$\mu_{1}/\mu_{0})$ was set to be 5. The computational domain and the initial state or steady deformed state of the RBC are shown in Figure 1. The problem was characterized by the capillary number (Ca),

(1) $$Ca=\frac{\mu_{0}\dot{\gamma }a}{G_{s}},$$

where $G_{s}$ is the surface shear elastic modulus, and $\dot{\gamma }$ (=$U_{m}^{\infty }/H$) is the shear rate defined by the mean velocity of the external fluid without cell $U_{m}^{\infty }$ and channel height *H* (=10 μm). Since the inertial effect can be negligible in the microfluidic device, we set Re as small enough to assume the Stokes flow. To reduce the computational costs, we set $Re=\rho U^{\infty }H/\mu_{0}$ = 0.2, where ρ is the external fluid density and $U^{\infty }$ is the maximum velocity of the external fluid with no cell. This value accurately represents the capsule dynamics solved by the boundary integral method in Stokes flow [12,33].

The membrane was modeled as an isotropic and hyperelastic material that followed the Skalak constitutive (SK) law [32]. The strain energy *w* and principal tensions in the membrane $T_{1}$ and $T_{2}$ ($T_{1}\geq T_{2}$) of the SK law are given by

(2) $$w=\frac{G_{s}}{4}\left(I_{1}^{2}+2I_{1}-2I_{2}+CI_{2}^{2}\right),$$

and

(3) $$T_{1}=\frac{G_{s}\lambda_{1}}{\lambda_{2}}\left[\lambda_{1}^{2}-1+C\lambda_{2}^{2}\left(\lambda_{1}^{2}\lambda_{2}^{2}-1\right)\right],\;\left(\mathrm{likewise}\;\mathrm{for}\;T_{2}\right),$$

where *C* is a coefficient representing the area incompressibility, $I_{1}(=$$\lambda_{1}^{2}+\lambda_{2}^{2}-2)$ and $I_{2}\;\left(=\;\lambda_{1}^{2}\;\lambda_{2}^{2}\;-\;1\;=\;J_{s}^{2}-1\right)$ are the first and second invariants of the strain tensor, $\lambda_{1}$, $\lambda_{2}$ are the two principal in-plane stretch ratios, and $J_{s}$ = $\lambda_{1}\lambda_{2}$ is the Jacobian, which expresses the ratio of the deformed to reference surface areas. If $I_{2}$ equals zero (i.e., $J_{s}$ = 1), the membrane satisfies perfect incompressibility. In this study, the surface shear elastic modulus and area incompressibility coefficient of RBCs were determined to be $G_{s}$ = 4.0 μN/m and *C* = 10${}^{2}$, respectively [6,33]. The bending resistance $k_{b}$ was also considered [36], with a bending modulus $k_{b}$ = 1.2 × 10${}^{-19}$ J, according to the order of the value of $k_{b}$ [37].

### 2.2. Numerical Simulation

**Figure 2:**
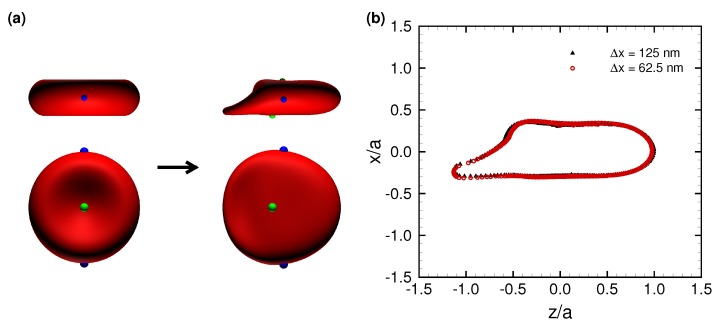
(**a**) Typical snapshots of a deformed RBC subjected to Ca = 0.15 at the initial state (left) and steady state (right). Two views, from the span-wise and stream-wise directions, are shown above and below, respectively. The markers represent node points. (**b**) Superposition of the fully deformed RBC projected on the *x*-*z* plane at Ca = 0.15. The two lines obtained with Δx = 125 nm (black) and 62.5 nm (red), respectively. The membrane position is normalized by the reference radius *a*.

We used the LBM [38] coupled with the finite element method (FEM) [39]. The membrane mechanics were solved by the FEM, and are given by

(4) $$\int_{S}\hat{u}\cdot qdS=\int_{S}\hat{\epsilon }:TdS,$$

where T is the Cauchy stress tensor, q is the load on the membrane, $\hat{u}$ is the virtual displacement, and $\hat{\epsilon }=\left(\nabla_{s}\hat{u}+\nabla_{s}{\hat{u}}^{T}\right)/2$ is the virtual strain tensor. The fluid mechanics were solved by the LBM [38] as,

(5) $$f_{i}\left(x+c_{i}\Delta t,t+\Delta t\right)-f_{i}\left(x,t\right)=-\frac{1}{\tau }\left[f_{i}\left(x,t\right)-f_{i}^{eq}\left(x,t\right)\right]+F_{i}\Delta t,$$

where $f_{i}$ is the particle distribution function for ideal particles with velocity $c_{i}$ at position x, Δt is the time step size, $f_{i}^{eq}$ is the equilibrium distribution, τ is the nondimensional relaxation time, and $F_{i}$ is the external force term. Subscript *i* represents the distribution direction of an ideal particle (*i* = 0–18). The D3Q19 LBM was used. FEM and LBM were coupled by the immersed boundary method [40]. All procedures were fully implemented on a GPU to accelerate the numerical simulation [41]. Our coupling method has been successfully applied to numerical analyses of cellular flow [33,34,35] and cell adhesion [12]. The solid and fluid mesh sizes were set to be 125 nm (an unstructured mesh with 20,480 elements was used for the RBC membrane). This resolution has been shown to successfully represent single-cell dynamics in a channel [12]. The results of cell deformation did not change with twice the fluid-mesh resolution (Figure 2b).

## 3. Results

### 3.1. Deformation of a Translating RBC in a Narrow Rectangular Microchannel

**Figure A2:**
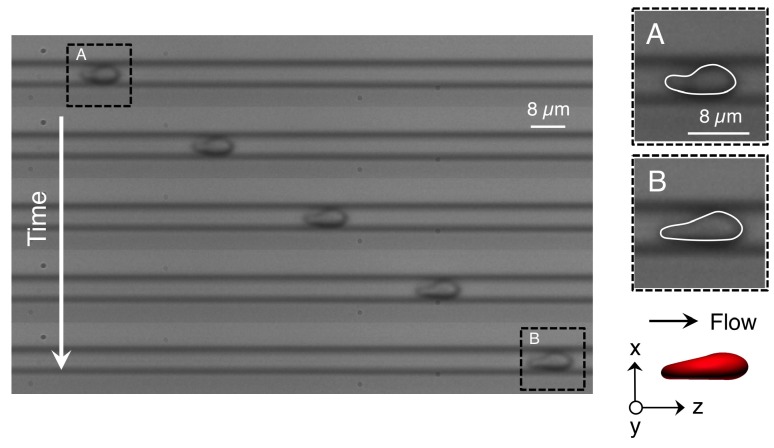
Representative images of the flowing RBC at frame numbers 0 (top), 20, 40 60, and 80 (bottom), respectively, where the speed of the cell is 1200 μm/s (see also Video S1). A representative numerical result of a stable slipper-shaped RBC subjected to Ca = 0.01 (calculated centroid velocity $V_{c}∼$ 25 μm/s) is also displayed (see also Video S2).

We performed numerical simulations to reproduce a translating RBC in a narrow rectangular microchannel, as shown in Figure A1d, and found that the RBC demonstrated an asymmetrical shape, the so-called slipper shape [42], which was also observed in the experiment as shown in Figure A2 (see also Videos S1 and S2). A typical asymmetrical shape of a deformed RBC subjected to Ca = 0.15 is shown in Figure 2a, where the markers represent membrane node points. The result clearly shows that the membrane does not rotate; in other words, the RBC stably translates without a tank-treading motion. The outlines of the deformed RBC at different fluid mesh resolutions are shown in Figure 2b, projected on the *z*-*x* plane. The result remains the same with twice the fluid mesh resolution (Δx = 62.5 nm). Therefore, the present resolution (Δx = 125 nm) successfully reproduces the fluid dynamics between the membrane and wall, and will be used in this study.

**Figure 3:**
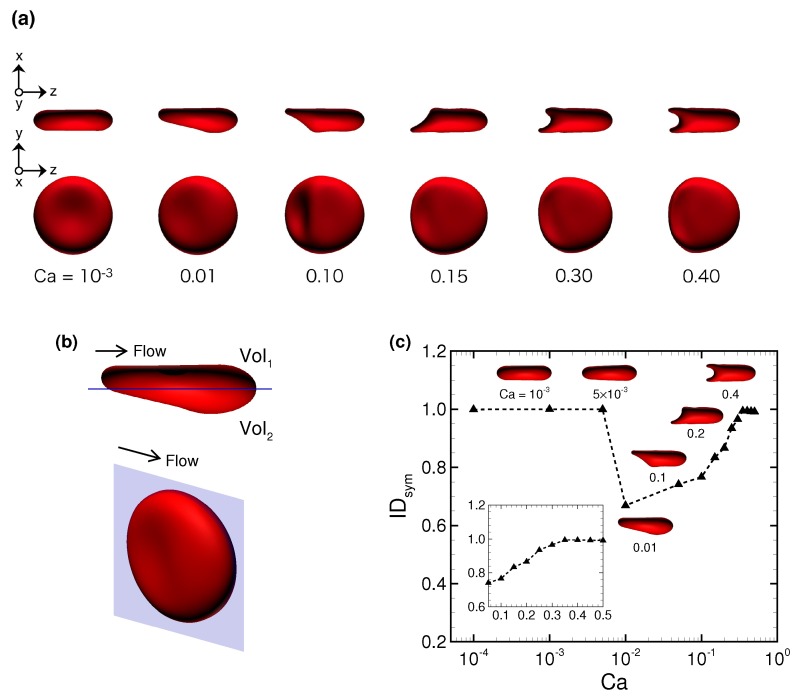
(**a**) Snapshots of a fully deformed RBC for different Ca. (**b**) Typical snapshots of a RBC at Ca = 0.01, where the blue plane denotes the center of the *x*-*z*-plane parallel to the flow direction, dividing the cell into the volume 1 ($Vol_{1}$) and volume 2 ($Vol_{2}$). (**c**) The symmetry index $ID_{sym}$ as a function of Ca. The insets represent snapshots of deformed RBCs at specific Ca.

Figure 3a shows snapshots of a stable RBC configuration for different Ca at fully developed flow. The RBC demonstrated an almost unchanged (symmetrical) biconcave shape at small Ca = 10${}^{-3}$, then shifted to an asymmetrical slipper shape as Ca increased (see also Video S3 for Ca = 0.1), and finally attained a symmetrical parachute shape at Ca≥ 0.35 (see also Video S4 for Ca = 0.5). To quantify the symmetry of the stable configuration of the deformed RBC, we propose a symmetry index $ID_{sym}$, which is defined by the volume ratio of two volumes that are divided by a plane parallel to the flow direction at the midline of the channel, as shown in Figure 3b. Using volume 1 ($Vol_{1}$) and volume 2 ($Vol_{2}$), $ID_{sym}$ is given as

(6) $$ID_{sym}=\frac{\mathrm{MIN}\left(Vol_{1},Vol_{2}\right)}{\mathrm{MAX}\left(Vol_{1},Vol_{2}\right)}.$$

A complete symmetrical shape is expressed as $ID_{sym}$ = 1. We show the results of $ID_{sym}$ as a function of Ca in Figure 3c. An asymmetrical parachute shape abruptly appeared for Ca≥ 0.01, but it gradually recovered and finally reached $ID_{sym}$ = 1 for Ca≥ 0.35. These results suggest that there exists the following specific range of Ca that allows a RBC to deform into an asymmetrical slipper shape: 5 × 10${}^{-3}<Ca<$ 0.35.

**Figure 4:**
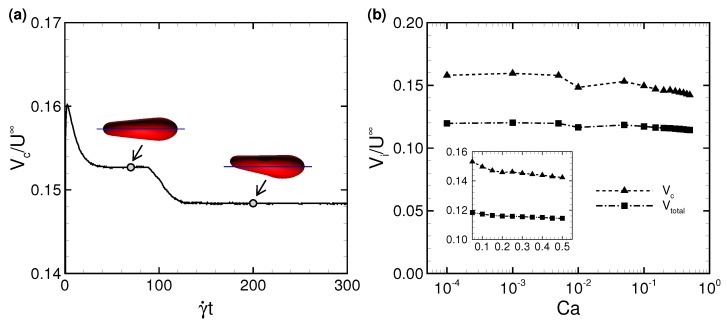
(**a**) Time history of the RBC centroid velocity ($V_{c}$) at Ca = 0.01, where $V_{c}$ = 24.7 μm/s at $\dot{\gamma }t$ = 200 corresponding to *t* = 12 s. The images represent snapshots of the deformed RBC at $\dot{\gamma }t$ = 70 (*t* = 4.2 s) and 200 (*t* = 12 s), respectively. The blue line denotes the center axis of the channel. (**b**) Time average of the RBC centroid velocity $V_{c}$ and total fluid velocity $V_{total}$ as a function of Ca. The velocity $V_{i}$ is normalized by the characteristic fluid velocity without cell $U^{\infty }$, where $V_{i}$ represents $V_{c}$ and $V_{total}$ by the index *i* = “c” or “total”.

Figure 4a shows one example of the temporal history of the RBC centroid velocity $V_{c}$ at Ca = 0.01, where $V_{c}$ is normalized by the characteristic (maximum) fluid velocity without cell $U^{\infty }$. The centroid velocity of RBC is calculated as a volume-averaged velocity, and is given by,

(7) $$V_{c}=\frac{1}{V}\int_{V}v\left(x_{m}\right)dV=\frac{1}{V}\int_{V}\nabla \cdot \left(v⊗r\right)dV=\frac{1}{V}\int_{S}n\cdot \left(v⊗r\right)dS,$$

and

(8) $$V=\int_{V}dV=\frac{1}{3}\int_{V}\nabla \cdot rdV=\frac{1}{3}\int_{S}n\cdot rdS,$$

where $v(x_{m})$ is the interfacial velocity of the membrane at the membrane node point $x_{m}$, r is the membrane position relative to the center of the RBC, n is the surface normal vector, V the volume of the RBC, and *S* is the surface area of the membrane. The velocity slightly (∼3%) decreased when the RBC shape changed from a symmetrical to asymmetrical shapes at Ca = 0.01 (Figure 4a). Because the membrane of a slipper-shaped RBC is dragged by the fluid near the wall, $V_{c}$ is slower than that of a symmetrically shaped RBC. Once the membrane deformed into an asymmetrical shape, that shape persisted. In this study, we defined the “steady state” as the condition wherein the centroid velocity reached a plateau (this time is hereafter referred to as $\dot{\gamma }t$ = 0), and used data after $\dot{\gamma }t$ = 0 to reduce the influence of the initial conditions. A time average was performed for the period $\dot{\gamma }t\geq$ 100 after $\dot{\gamma }t$ = 0. Figure 4b shows the time average of centroid velocity $V_{c}$ and total fluid velocity $V_{total}$ for different Ca, where those values are normalized by characteristic velocity $U^{\infty }$. The tendency that $V_{c}$/$U^{\infty }$ slightly decreases as Ca increases (Figure 4b) agrees with previous numerical results of a spherical capsule in a square channel [43] and constricted channel [44]. Note that the dimensional cell velocity basically increases as Ca increases, for instance, $V_{c}∼$ 0.12 μm/s for the lowest Ca (=10${}^{-4}$) and $V_{c}∼$ 1200 μm/s for the highest Ca (=0.5).

**Figure 5:**
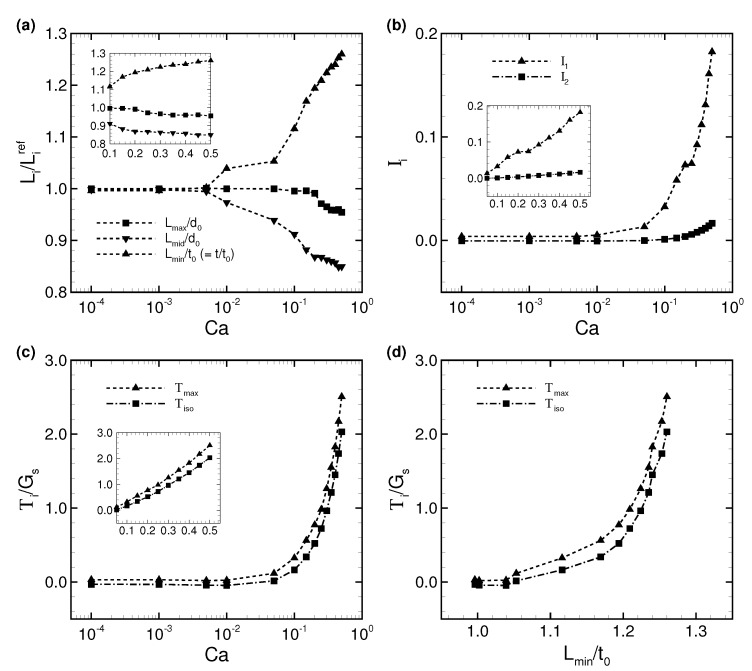
(**a**) Time average of the deformation index $L_{i}/L_{i}^{ref}$ as a function of Ca, where the maximum, middle, and minimum axis lengths ($L_{max}$, $L_{min}$, and $L_{mid}$, respectively) are normalized by each reference length $L_{i}^{ref}$ (i.e., no flow condition), where the reference major and minor axis lengths are $d_{0}$ and $t_{0}$ (thickness), respectively. (**b**) Averaged first and second invariants $I_{i}$ (*i* = 1 and 2) as a function of Ca. (**c**) Averaged maximum and isotropic tensions; $T_{max}$ and $T_{iso}$, respectively. These values are normalized by the shear elastic modulus $G_{s}$. (**d**) The average of these tensions, $T_{i}$, as a function of the deformation index $L_{min}/t_{0}$, which is the ratio between the minimum axis length of the deformed RBC (thickness) and the reference thickness.

The deformation of each axis in a steady-state membrane is quantified by the deformation index $L_{i}/L_{i}^{ref}$, which is the ratio between each axis length of a deformed RBC $L_{i}$ and each reference axis length $L_{i}^{ref}$ (i.e., without flow), where subscript *i* represents the maximum, middle and minimum axes (i.e., *i* = “max”, “mid”, and “min”). The results of $L_{i}/L_{i}^{ref}$ are shown in Figure 5a. We found that only the minimum axis (i.e., thickness) increases as Ca increases, while the maximum and middle axes decrease.

To quantify the strain of an isotropic elastic membrane, the first and second invariants of the strain tensor $I_{i}$ (*i* = 1 and 2) are calculated, and are given in Figure 5b. These are averaged by the total number of membrane meshes and the analysis duration, i.e.,

(9) $$I_{i}=\frac{1}{TS}\int_{t}\int_{S}I_{i}\left(x_{g},t\right)dSdt\;\left(i=1\;\mathrm{and}\;2\right),$$

where T is the period of analysis duration, and $x_{g}$ is the centroid of the triangle element of the membrane. According to Figure 5b, the second invariant $I_{2}$ is almost zero for Ca≤ 0.1, and only slightly increases for Ca> 0.1. Therefore, the membrane incompressibility is well maintained even after the membrane demonstrates the slipper/parachute shape. The first invariant $I_{1}$, on the other hand, starts to increase from Ca≥ 0.01 and grows rapidly compared to $I_{2}$. Therefore, the symmetrical parachute-like deformation results from greater membrane extension than the asymmetrical slipper-like deformation.

We also investigated the maximum in-plane principal tension $T_{max}$ ($T_{1}\geq T_{2}$) and the isotropic tension $T_{iso}(=$$T_{1}+T_{2})$/2 in the deformed RBC, and show the results in Figure 5c. We calculated the average value of those tensions as $T_{max}$ and $T_{iso}$ by using Equation (9). As expected, both tensions start to increase simultaneously when $I_{1}$ increases (i.e., Ca = 0.01). The isotropic tension $T_{iso}$ is always lower than the maximum principal tension $T_{max}$. To demonstrate the relationship between tension and deformation, $T_{i}$ is described as a function of the deformation index $L_{min}/t_{0}$ in Figure 5d. The result clearly shows the strain-hardening behavior of the RBC due to the nonlinearity of the SK law.

### 3.2. Effects of Perturbations on Stable Membrane Configuration

**Figure 6:**
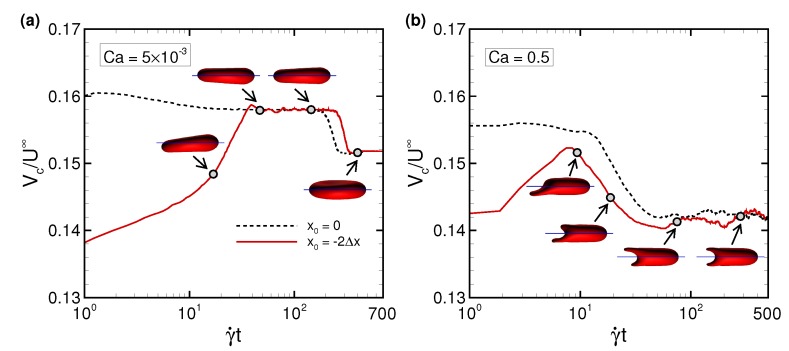
Time history of the RBC centroid velocity ($V_{c}$) at (**a**) low Ca = 5 × 10${}^{-3}$, and (**b**) high Ca = 0.5 for different initial positions along the span-wise direction $x_{0}$, where one RBC is initially placed at the midline of the channel ($x_{0}$ = 0, dashed line) and the other RBC is placed two fluid meshes away from the midline ($x_{0}$ = −2Δx, red line). The images represent snapshots of the RBC with $x_{0}$ = −2Δx at the indicated times (see also Videos S5 and S6). Note that $V_{c}$ = 12.7 μm/s for Ca = 5 × 10${}^{-3}$ at $\dot{\gamma }t$ = 500 (*t* = 60 s), and $V_{c}$ = 1180 μm/s for Ca = 0.5 at $\dot{\gamma }t$ = 500 (*t* = 0.6 s).

**Figure 7:**
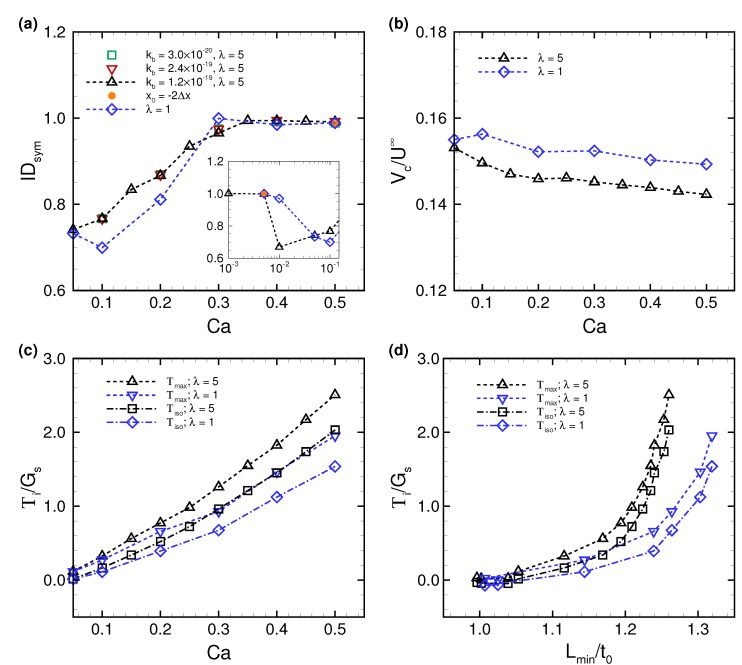
(**a**) The symmetry index $ID_{sym}$ as a function of Ca for different values of bending modulus $k_{b}$ = 3.0 × 10${}^{-20}$ J (square), 1.2 × 10${}^{-19}$ J (triangle), and 2.4 × 10${}^{-19}$ J (inverted triangle). The results obtained with a viscosity ratio of unity (i.e., λ = 1) are also displayed (diamond). The circular dot represents the result of $x_{0}$ = −2Δx at low Ca (= 5 × 10${}^{-3}$) and high Ca (= 0.5) and λ = 5. These results were obtained with $k_{b}$ = 1.2 × 10${}^{-19}$ J. (**b**) Time average of the RBC centroid velocity ($V_{c}$) as a function of Ca for different viscosity ratios (λ = 1 and 5). (**c**) Averaged maximum and isotropic tensions, $T_{max}$ and $T_{iso}$, respectively. (**d**) Averaged tension $T_{i}$ as a function of the deformation index $L_{min}/t_{0}$.

To clarify the reproducibility of the stable configuration of a deformed RBC in the narrow rectangular microchannel, we investigated the effects of potential perturbations, e.g., the initial centroid position $x_{0}$, bending rigidity $k_{b}$, and viscosity ratio λ. Figure 6 shows the centroid velocity $V_{c}$ of a RBC subjected to low Ca (= 5 × 10${}^{-3}$) and maximum Ca (= 0.5) for different initial centroid positions along the span-wise direction of the channel. When the centroid of the RBC was initially placed two fluid meshes away from the midline of the channel (i.e., $x_{0}$ = −2Δx), the RBC started to flow with an asymmetrical slipper shape, but gradually migrated to the channel axis due to the lift forces induced by the wall and shear gradient, and finally attained a symmetrical shape for both Ca values with the same velocity as that obtained with $x_{0}$ = 0 (Figure 4; see also Videos S5 and S6). Therefore, the stable configuration of the deformed RBC is insensitive to the initial position. Note that although the RBC subjected to low Ca (= 5.0 × 10${}^{-3}$) did not perfectly orient parallel to the flow direction (Figure 6a) and suffered from decreasing the cell velocity, the symmetry index $ID_{sym}$ remained the same (Figure 7a).

We also tested different values for bending rigidity $k_{b}$, where the value of $k_{b}$ was set to a quarter of the original bending resistance ($k_{b}$ = 3.0 × 10${}^{-20}$), and twice the original bending resistance ($k_{b}$ = 2.4 × 10${}^{-19}$). As shown in Figure 7a, the symmetry index $ID_{sym}$ remained same regardless of the value of $k_{b}$. Therefore, bending rigidity does not affect the stable configuration of the translating RBC in the narrow rectangular microchannel, at least within the parameter space that we investigated, namely 3.0 × 10${}^{-20}\leq k_{b}\leq$ 2.4 × 10${}^{-19}$.

However, $DI_{sym}$ was affected by the viscosity ratio λ. When λ decreased to unity (i.e., λ = 1), the membrane tended to assume a symmetrical shape even at relatively low Ca = 0.01. The most asymmetrical shape was found at at λ = 5, and the minimum $DI_{sym}{|}_{\lambda =1}$ shifted to larger Ca≈ 0.1 (Figure 7a). The value of $DI_{sym}$ at λ = 1 started to recover beginning at Ca = 0.1, and finally almost reached 1 at Ca = 0.3. To see the effect of λ, we compared the centroid velocity $V_{c}$ and membrane tension $T_{i}$ between different λ (= 1 and 5). $V_{c}$ at λ = 1 tended to be larger, and was approximately 4% greater than that obtained with λ = 5 (Figure 7b). The results of $T_{i}$, on the other hand, tended to decrease as λ decreased (Figure 7c).

Figure 7d shows the membrane tensions as a function of the deformation index $L_{min}/t_{0}$. When $L_{min}/t_{0}$ was invariant, the tensions acting on the membrane $T_{i}$ tended to be lower as λ decreased. This tendency was inconsistent with the previous numerical results of the RBC in simple shear flow [45]. Compared with the previous results in [45], the similarities or discrepancies in the values of $T_{i}$ (Figure 7c,d) for different λ would arise from different flow modes and confined geometries. Even though tensions acting on the membrane and deformation depend on λ, the RBC in the narrow rectangular microchannel underwent the same history of deformation as a function of Ca; the almost original biconcave shape at low Ca, and an asymmetrical slipper shape at low/moderate Ca, and finally a symmetrical parachute shape at high Ca. These results suggest that the stable configuration of the translating RBC in the narrow rectangular microchannel was reproducible independently of any perturbations that we investigated.

## 4. Discussion

The asymmetric slipper shape of RBCs has been found in capillaries [42], and the motion has been systematically investigated both in experiments [19,20,21,22,23] and in numerical simulations [20,26,28,30]. An experiment using microfluidic devices showed that RBCs undergo a transition from a symmetrical parachute shape to an asymmetrical slipper shape as cell velocity increased [23]. Other experimental results showed that viscous shear stresses controlled this transition, and confinement was not necessary for the slipper shape [19]. The results reported in [19] are consistent with the numerical results obtained using a two-dimensional (2D) droplet model [26]. The numerical studies reported in [26] clearly showed that the shape transition in an unbounded Piseuille flow occurred when a dimensionless vesicle deflation number, representing shape stability, fell below a certain value. Other numerical results reported in [28] demonstrated that 2D droplets also assumed the slipper shape, not only in an unbounded Piseuille flow but also in a confined channel. These numerical studies also clarified the effect of the viscosity ratio λ on stable configuration, showing specifically that a droplet with λ = 1 transitioned from a parachute shape to a slipper shape as the flow strength decreased [26], while a droplet with λ≈ 5 made this same transition as the flow strength increased [28]; these findings were consistent with the experimental results reported in [23]. Since the above numerical analyses were performed using a 2D droplet model, it is uncertain whether their results are applicable to the dynamics of a translating three-dimensional (3D) RBC in a confined rectangular channel when taking membrane elasticity into account. Fedosov et al. (2014) systematically investigated the behavior of single RBC in cylindrical microchannels for a wide range of channel confinements (2a/D, being a channel diameter *D*) using a 3D dissipative particle dynamics model [30], but the cross-sectional area of the microchannels were relatively large (2a/D< 0.8) comparing to a narrow rectangular microchannel represented in [17], where the channel confinement is characterized as 2a/H = 0.8 and 2a/W∼ 2.29 using the wall-normal length *H* and span-wise length *W* (Figure A1a–c). We thus numerically investigated the behavior of translating RBCs in a narrow rectangular microchannel that mimicked a microfluidic device (Figure A1) [17] with different Ca. Our numerical results demonstrated that the confined RBCs maintained a nearly unchanged, biconcave shape at low Ca, then shifted to an asymmetrical slipper shape at low/moderate Ca, and finally attained a symmetrical parachute shape at high Ca. Such asymmetrical slipper shape was also observed in the experiment (Figure A2). The finding that RBCs tended to show a symmetrical shape with increasing Ca contradicted previous experimental results [23] as well as numerical results obtained using a 2D-droplet model with λ≈ 5 [28]. This discrepancy may have been caused by the effects of three-dimensional flow structures in a confined channel and by the membrane constitutive law. Our numerical results of the transition from slipper to parachute shapes qualitatively agree with those obtained in cylindrical microchannels for 2a/D< 0.8 [30]. To the best of our knowledge, such shape transition in a narrow rectangular microchannel that was presented here is the first of its kind. We also showed that the stable configuration of the translating RBC in the narrow rectangular microchannel was reproducible independently of any perturbations that we investigated such as the initial centroid position, bending rigidity, and viscosity ratio. If the fully deformed configuration or the transition mode is related to membrane shear elasticity, which is characterized by Ca, these insights will help us identify the cell state. Since different motions of individual RBCs may affect the bulk suspension rheology [46], identifying a stable mode of RBCs in a channel will be also helpful to evaluate the blood rheology.

In our experiment using microfluidic devices, we observed a slipper-shaped RBC whose velocity was almost 1.2 mm/s (Figure A2), while the numerical results showed that a velocity this high resulted in a RBC with a symmetrical parachute shape (Figure 3c). This discrepancy may have been due to the duration of the observation. Since experimental observation periods are limited to 0.1 s or less, the slipper-shaped RBC in the microfluidic device may have been in the transition. According to our numerical results shown in Figure 6, the transition from slipper shape to parachute shape takes at least $\dot{\gamma }t∼$ 300, corresponding to ∼0.3 s for Ca = 0.5 ($V_{c}∼$ 1.2 mm/s). Another possible reason may have been due to RBC heterogeneity as reported in our previous experiments [17,18]. The experimental observations of an asymmetrical slipper shape in a microfluidic device are required for precise statistical analysis, which will be addressed in future study.

We are not sure what perturbations are needed to destroy the stable symmetrical shape. Thermal energy is unlikely to be affecting the state: indeed, although the RBC membrane usually demonstrates Brownian motion in the free state, the Peclet number ($Pe=\dot{\gamma }a/D_{p}$, being a radius of the RBC *a* and a diffusion coefficient $D_{p}$) is estimated as approximately O(Pe) = 10${}^{1}$, even at Ca = 10${}^{-4}$, by using the Stokes–Einstein equation, and thus the Brownian diffusion (thermal fluctuations) should have little effect. Although the membrane bending rigidity did not affect the stable membrane configuration at least for 3 × 10${}^{-20}$ J ≤Ca≤ 2.4 × 10${}^{-19}$ J, further investigation will be required for larger parameter spaces. In this study, we defined the initial shape of RBCs as a biconcave disc. Since some recent numerical studies have debated the stress-free shape of RBCs [47,48,49], it will be interesting to study how the reference shape (biconcave, oblate spheroid, and sphere) affects the stable configuration of translating RBCs in a narrow rectangular microchannel.

## 5. Conclusions

We numerically investigated the dynamics of translating RBCs in a narrow rectangular microchannel for different capillary numbers (Ca). Our numerical results demonstrated that a confined RBC in a narrow rectangular microchannel maintained a nearly unchanged, biconcave shape at low Ca, then assumed an asymmetrical slipper shape at moderate Ca, and finally attained a symmetrical parachute shape at high Ca. Once the RBC deformed into either of the latter two shapes, they sustained that shape as their final stable configurations. The membrane deformation as a function of Ca remained the same even when the viscosity ratio λ decreased from physiological relevant value (λ = 5) to unity. The final stable configuration was insensitive to bending resistance and initial position. If these shapes are found in diseased RBCs translating at specific velocities, the shapes will be an important indicator of cell state.

## Supplementary Materials

Following materials are available at https://www.mdpi.com/2072-666X/10/3/199/s1, Video S1: experimental result at the speed of the cell being 1200 μm/s, corresponding to Ca≈ 0.5; Video S2: numerical result at Ca = 0.01; Video S3: numerical result at Ca = 0.1; Video S4: numerical result at Ca = 0.5; Videos S5 and Video S6: numerical results of initial RBC centroid position two meshes away from the midline for Ca = 5 × 10${}^{-3}$ and Ca = 0.5, respectively. These numerical results were obtained with λ = 5 and $k_{b}$ = 1.2 × 10${}^{-19}$ J.

## Abbreviations

The following abbreviations are used in this manuscript:

RBC	Red blood cell
LBM	Lattice–Boltzmann method
FEM	Finite element method
IBM	Immersed boundary method
GPU	Graphics processing unit

## Appendix A. Sample Preparation and Observation

Adult blood was drawn from healthy donors based on the informed consent. All the experiments and experimental protocols in microchannels were approved by the Ethical Committee of Osaka University and performed according to the appropriate guidelines and regulations. Immediately after the blood was drawn, it was maintained in an intact condition by dispersal at a concentration of 1% (v/v) in standard saline.

A microfluidic channel was constructed between a glass slide and poly (dimethylsiloxane) (PDMS) that was designed and printed from a master mold made of SU-8 photoresist using standard photolithography. The cross-section of the rectangular microchannel was 10 μm × 3.5 μm (Figure A1a,b). The experimental system was composed of a high-speed camera (IDP-Express R2000, Photron) and microscope (IX71, Olympus) equipped with an × 40 (N.A. = 0.6) or × 50 (N.A. = 0.42) objective lenses. Images were captured at 1000 frames/s with exposure time of 1 ms. The spatial resolutions of captured images were 0.24 μm/pixel (Figure A1d) and 0.26 μm/pixel (Figure A2) for × 40 and × 50 objective lenses, respectively. Flow of the solution inside the microchannel was basically driven by a constant pressure difference between the inlet and outlet of the channel, maintained by atmospheric pressure and gravitational force.

Figure A2 shows representative snapshot images of a RBC flowing at a velocity of 1200 μm/s; imaging was performed at 1000 frames/s, perpendicular to the span-wise direction of the microchannel. The RBC deformed into an asymmetrical shape, the so-called slipper shape [42]. Interestingly, this configuration was also found in a numerical simulation with Ca = 0.01, where the RBC centroid velocity was $V_{c}∼$ 25 μm/s (Figure A2). Although we reported RBC heterogeneity in [17], the asymmetrical shape of RBCs by means of the experimental observations is required for a precise statistical analysis, which is however future study.
